# Strengthening Kampala’s Urban Referral System for Maternal and Newborn Care Through Establishment of an Emergency Call and Dispatch Center

**DOI:** 10.9745/GHSP-D-22-00332

**Published:** 2023-06-21

**Authors:** Sam Ononge, Andrew Magunda, Dorothy Balaba, Peter Waiswa, Daniel Okello, Henry Kaula, Sara Zalwango, Douglas Akii Bua, Amable Ayebare, Frank Kaharuza, Cudjoe Bennett, Sara Sulzbach, Brett Keller, Yvonne Mugerwa

**Affiliations:** a Makerere University College of Health Sciences, Kampala, Uganda.; b Population Services International Uganda, Kampala, Uganda.; c Kampala Capital City Authority, Kampala, Uganda.; d Kampala University, Kampala, Uganda.; e Office of Maternal and Child Health and Nutrition, Bureau of Global Health, U.S. Agency for International Development, Washington, DC, USA.

## Abstract

**Introduction:**

Most pregnant women living in urban slum communities in Uganda deliver at public health centers that are not equipped to provide emergency obstetric and newborn care. When obstetric emergencies occur, pregnant women are referred to a higher-level facility and are responsible for arranging and paying for their own transport. The Kampala Slum Maternal Newborn (MaNe) project developed and tested an emergency call and ambulance dispatch center and a mobile application to request, deploy, and track ambulances. We describe the development of these 2 interventions and findings on the feasibility, acceptability, and sustainability of the interventions.

**Methods:**

MaNe conducted a mixed-method feasibility study that included an assessment of the acceptability and demand of the interventions. In-depth interviews (N=26) were conducted with facility proprietors, health providers, ambulance drivers, Kampala Capital City Authority officers, and community members to understand the successes and challenges of establishing the call center and developing the mobile application. Thematic content analysis was done. Quantitative data from the call center dispatch logs were analyzed descriptively to complement the qualitative findings.

**Findings:**

Between April 2020 and June 2021, 10,183 calls were made to the emergency call and dispatch center. Of these, 25% were related to maternal and newborn health emergencies and 14% were COVID-19 related. An ambulance was dispatched to transfer or evacuate a patient in 35% of the calls. Participants acknowledged that the call center and mobile application allowed for efficient communication, coordination, and information flow between health facilities. Supportive district leadership facilitated the establishment of the call center and has taken over the operating costs of the center.

**Conclusion:**

The call center and referral application improved the coordination of drivers and ambulances and allowed facilities to prepare for and treat cases more efficiently.

## Abstract (precis)

An emergency call and ambulance dispatch center facilitated maternal and newborn transport, and a smartphone application helped deploy and track ambulances to improve coordination and efficiency in emergency case referral and transport.

## Teaser

**Key Findings:**

- The Kampala Slum Maternal and Newborn Health project established an emergency call and ambulance dispatch center that improved coordination, efficiency, and accountability in the referral and transport of maternal and newborn health emergency cases to higher-level facilities.
- The project also developed and tested a smartphone application for initiating referral, tracking, and deployment of ambulances. The mobile application was considered an effective and feasible solution for deploying and tracking ambulances and improved driver accountability as locations could be viewed in real time.
- The mobile application ensured receiving referral facilities were notified, which improved their preparation and management of referral cases.

**Key Implication:**

- Involvement of the district authority and leadership in the planning, designing, and implementation of the intervention may improve ownership, acceptability, and sustainability of the project.

## INTRODUCTION

Globally, approximately 287,000 women died in 2020 due to complications during pregnancy and childbirth, and 206,000 of all maternal deaths and 2.5 million neonatal deaths occurred in sub-Saharan Africa.^1^^,^^2^ In Uganda, although the maternal mortality ratio has declined by 38.7% between 2000 and 2020, the rate (287 maternal deaths per 100,000 live births) is still higher than expected, as per the Sustainable Development Goal 3.1 target for the country.^1^ Evidence shows that, in most cases, 50% of obstetric maternal deaths could be averted through the provision of emergency obstetric care by skilled providers.^3^ According to the 2016 Uganda Demographic and Health Survey, 74% of childbirths happen with a skilled attendant.^4^ However, 56% of these childbirths happened in primary health centers^5^ that are inadequately equipped to provide emergency obstetric care services. Thus, there is a need for emergency transportation for referral to higher-level facilities when complications arise during delivery.^6^ Unfortunately, transport for referral to higher-level facilities that provide emergency obstetric care services is often hindered by ambulance shortages, lack of an ambulance transport system, personal financial constraints, and uncoordinated health systems.^7^^,^^8^ As such, women and their families are often required to arrange and pay for their own transport in emergency situations during childbirth.^9^^–^^11^

The delay in reaching appropriate obstetric care caused by health system factors contributes to an increased risk of maternal death,^12^^–^^14^ explained in the 3-delay model of Thaddeus and Maine.^15^ To mitigate this risk, a well-coordinated interfacility transfer of patients with obstetric and neonatal complications from low-tier health centers to higher-level facilities has been shown to reduce delays in receiving timely and appropriate care after reaching the referral facility (delay 3).^12^^,^^16^ A well-coordinated referral system includes prompt arrangement of transport, pre-referral stabilization of the patient, communication with the referral facility to prepare for the emergency, care during the referral, appropriate transfer at arrival, and feedback to the referring facility.^8^ Smooth coordination of these activities is a challenging task in most low-resource settings because of the lack of referral guidelines and protocols. Therefore, having an emergency call and dispatch center (ECDC) to coordinate these activities is a critical but challenging innovative solution in low-resource settings.

Several interfacility transportation interventions for pregnant women and newborns with complications have been implemented to address barriers to reaching facilities for emergency care in sub-Saharan African countries. These interventions include locating ambulances at the referring or referral facilities and using private ambulance service providers, motorbike “ambulances,” public commercial transport, and personal transport, including motorbikes.^8^^,^^17^^,^^18^ Despite many successful emergency transport interventions for maternal and newborn complications in low-resource settings,^17^^,^^19^^–^^23^ few have established an ECDC to facilitate coordination and communication between facilities.^8^^,^^24^^,^^25^ Review of the literature suggests that the establishment of emergency call centers with obstetric transportation and complementary interventions may reduce the rate of adverse pregnancy outcomes and increase access to skilled services for women in low- and middle-income countries.^6^ However, there is a paucity of high-quality studies, with many resource-constricted settings lacking organized emergency medical services due to inadequate training and financial constraints.^26^^–^^28^

Despite many successful emergency transport interventions for maternal and newborn complications in low-resource settings, few have established an emergency call and dispatch center to facilitate coordination and communication between facilities.

Uganda’s health care system is organized in a tiered manner that includes primary health care facilities, general hospitals, regional referral hospitals, and national referral hospitals. According to the Ministry of Health’s (MOH) basic package of services, designated services are provided at each level of care, with higher-level facilities providing services for more complex cases. It is assumed that this structure ensures that every higher level has a greater capacity to provide care. When complications require transfer to higher-level facilities, the country does not have structured protocols for coordinating referral processes.^29^ Furthermore, referral mechanisms are characterized by a lack of referral forms, nonfunctional feedback loops, and poor infrastructure for the movement of emergency vehicles.^30^ These challenges result in delays in emergency transportation, which, consequently, can affect patient outcomes. There is also no functional emergency communication system, which makes it difficult to provide ambulances to those who need them.^12^

Like many cities, migration to Kampala city (an urban setting) for economic reasons has resulted in a growth of slums and unplanned settlements. This trend has had negative consequences on access to emergency health services for the urban poor.^12^ According to the United Nations report on urbanization, the population of Kampala City increased from approximately 1.2 million in 2000 to 3.0 million in 2018.^31^ Similar to other low-resource settings, local populations often bypass seeking care at primary-level facilities for higher-level referral facilities based on illness recognition and facility-specific services.^12^^,^^32^ As a result, primary-level facilities are underutilized, and referral facilities are overcrowded.

The public health system in Kampala is managed by the Kampala Capital City Authority (KCCA), which is responsible for providing health care services to all residents at no cost. KCCA has oversight of both public and private facilities, which includes developing and implementing standards of care protocols and ensuring that all providers are licensed and maintain their credentials. A 2017 audit of health facilities in Kampala reported a total of 444 health facilities in Makindye and Rubaga; 5 were public and 439 were private facilities. In 2020, the KCCA had 6 public ambulances, an inadequate number to serve 3.0 million people. However, there were also many undocumented, unregulated, and underutilized privately owned ambulances, which can potentially be harnessed into a unified system in collaboration with public ambulances to serve the urban population more efficiently.

The Kampala Slum Maternal and Newborn Health (MaNe) Project, a collaboration between Population Services International Uganda and KCCA that was funded by the U.S. Agency for International Development (USAID), tested an innovative approach to address referral challenges in the urban slum communities of Kampala City. Using an implementation research approach, the MaNe project developed and tested an ECDC in Kampala City to strengthen and coordinate maternal and newborn health (MNH) referrals between facilities for timely and effective emergency care and a mobile application to deploy and track ambulances. The study aimed to assess: (1) the feasibility, acceptability, and sustainability of establishing an ECDC to facilitate emergency referrals; and (2) the feasibility and acceptability of a mobile application for initiating the referral, deployment, and tracking of ambulances and for providing feedback.

## METHODS

### Study Setting

**FIGURE 1:**
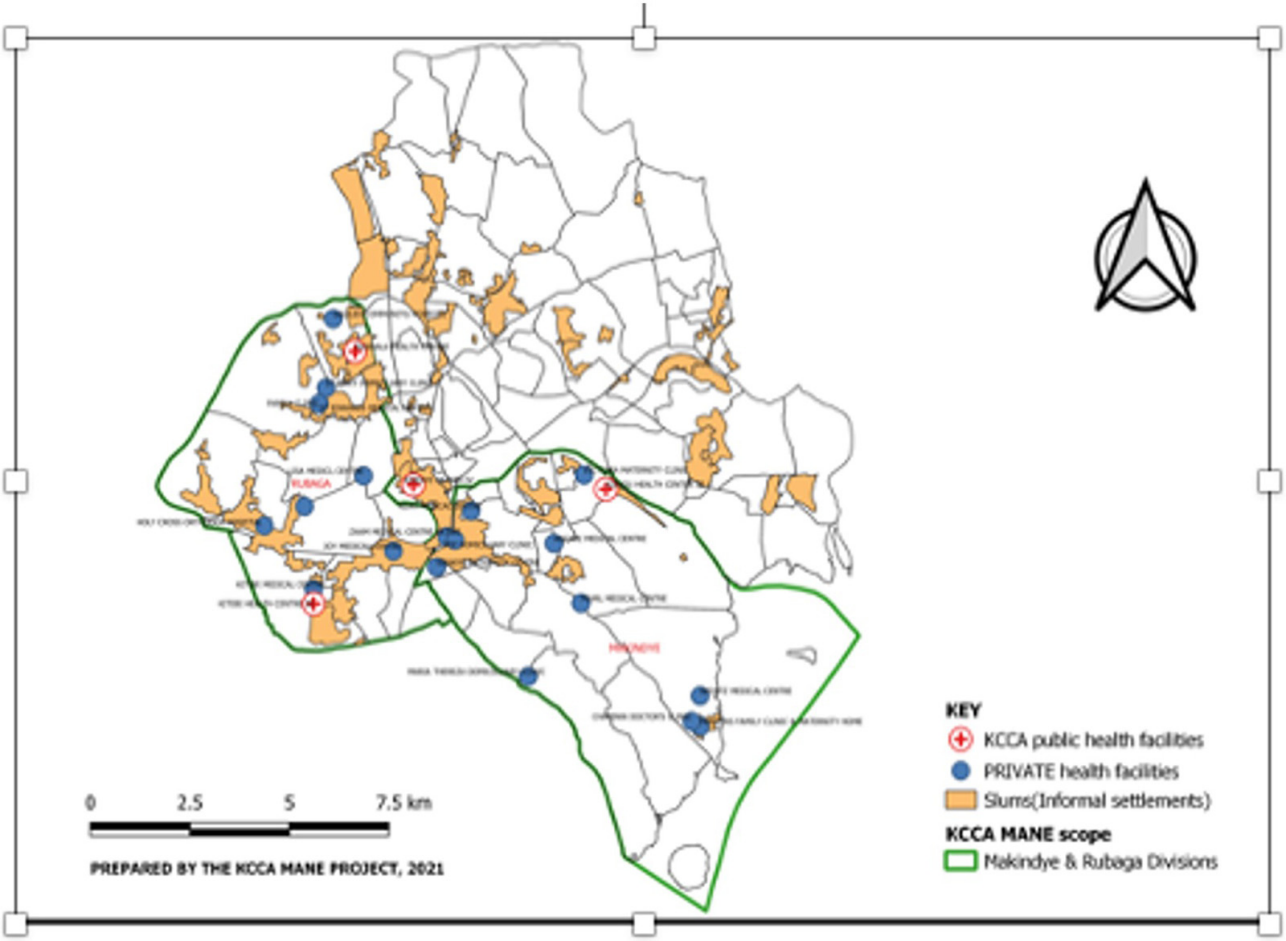
Map of Kampala City, Showing Locations of Informal Settlements, Public Facilities, and Private Facilities

Kampala City, located in the southern part of Uganda, is divided into 5 administrative divisions: Kampala Central, Kawempe, Nakawa, Makindye, and Rubaga. The MaNe project was implemented in Makindye and Rubaga divisions, which were purposively selected based on population density and the presence of informal settlements or slum communities (Figure 1). Makindye and Rubaga divisions host 60% of all informal settlements in Kampala. Makindye division has 15 informal settlements, while Rubaga division has 13.

The MaNe project was implemented in 4 public comprehensive emergency obstetric and newborn care facilities, 20 privately owned facilities, and 1 national referral hospital. Before the project, each of the government public facilities had their own ambulances and drivers and had a budget allocated for fuel and ambulance maintenance. In contrast, private facilities most often relied on patients’ relatives to arrange for transport for referral. The health provider’s availability to accompany the patients during referral depended on the number of staff at the facility at that particular time. Most patients were transported unaccompanied. KCCA had a call center to receive inquiries that was staffed by 1 person and only operated when he was on duty.

Before the intervention, each public facility used their own ambulance driver and private facilities relied on patients’ relatives for transport.

### Interventions

The interventions were ECDC and an interfacility ambulance mobile application called the Kampala Digital Emergency Transport System (KDETS). The ECDC had computers, phones, headsets, ambulance tracking screens, furniture, and Internet connectivity and was staffed by call center agents.

**FIGURE 2:**
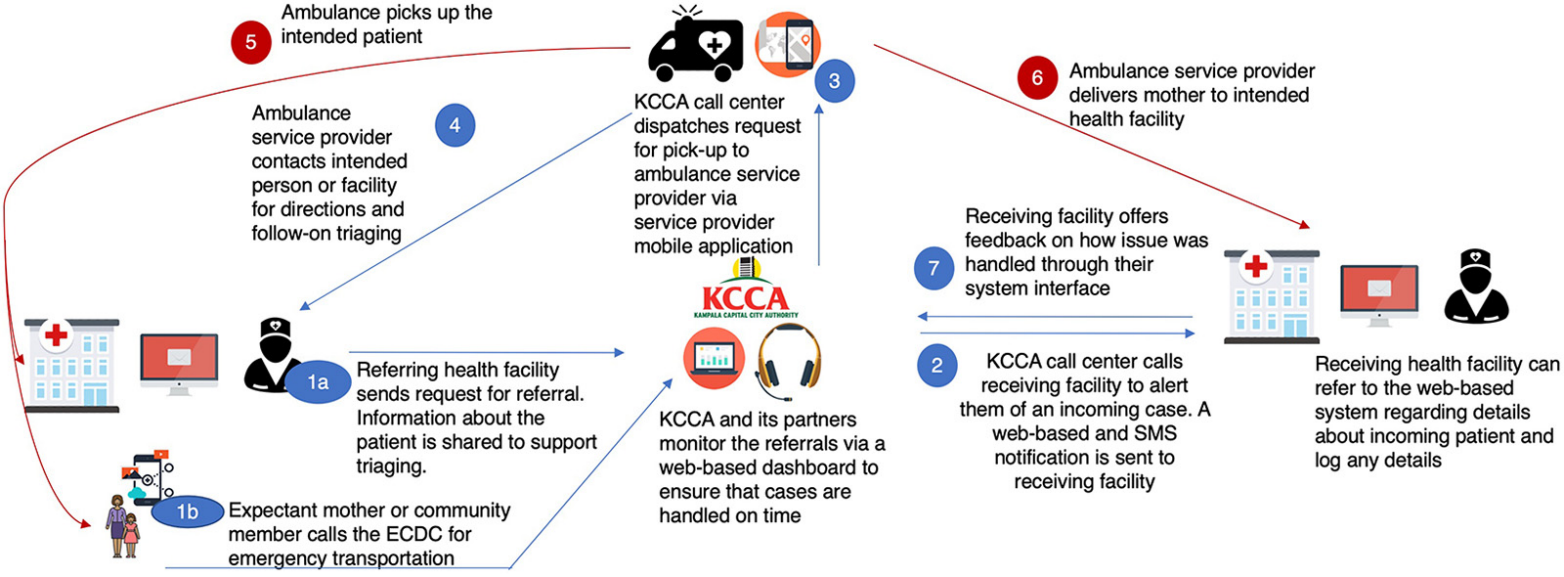
Flow From the Person Requesting an Ambulance Through the ECDC to the Ambulance Driver and the Referral Facility Abbreviations: ECDC, emergency call and dispatch center; KCCA, Kampala Capital City Authority.

The KDETS application had several uses: (1) private and public health facilities and the community could request referral transport; (2) requests for ambulances could be communicated through the ECDC; (3) ECDC agents dispatched the pickup request to available ambulance drivers; (4) ECDC agents notified providers at the receiving facility of a referred case to alert them to prepare; (5) after transporting the patient to the facility, ambulance drivers could add information on the transport to help monitor data on response time; and (6) health care providers could communicate case management information to the referring facilities (Figure 2).

### Study Design

The Consolidated Framework for Implementation Research^33^ was used to evaluate the establishment and operation of an ECDC and the development of the mobile application for tracking and deployment of ambulances in Kampala City. The design of the study instruments used to measure the outcome of the complex intervention was based on the domains of Consolidated Framework for Implementation Research, which offers a systematic approach that promotes the development of theory and verification of the target and scope of the intervention strategy.

### Preparatory Phase

The MaNe project began with formative research on MNH in Kampala. To respond to challenges discovered during the formative research phase, the MaNe project applied Population Services International’s Empathy, Insights, and Prototyping (EIP) approach to understand the needs of the target population and generate feasible and acceptable interventions.^34^ Details of each phase of EIP are in a Supplement.

During the empathy phase of the EIP process, project staff interacted with intended users of the intervention and public health leadership (e.g., KCCA and MOH) to design an intervention that was acceptable and feasible. This phase included conducting qualitative interviews with key stakeholders in the study sites and secondary data analysis of key MNH indicators.

During the insights phase, the project staff synthesized learnings from the empathy phase to understand the drivers of health behaviors and the needs of end users. This phase included 2 workshops: a data interpretation workshop and a cocreation workshop. KCCA leadership (technical and political) was involved in both workshops.

Finally, during the prototyping phase, the proposed solutions that emerged from the cocreation workshop were reviewed by the project design team and arranged thematically. These solutions, or prototypes, were evaluated for relevance and feasibility based on project objectives. Prototypes were tested by showing a schematic representation of the solutions to community members and soliciting their feedback about the acceptability, feasibility, and effectiveness of the proposed packages. A rapid prototyping approach was used, progressing from lower-fidelity representations of the interventions to higher-fidelity ones, with feedback collected by program staff throughout the process. This process resulted in a final package of interventions that included establishing the ECDC and the development of a ride-hailing service like Uber to facilitate the deployment and tracking of ambulances. The first prototype of the application drew on lessons from similar ambulance coordination applications, such as the Uber transport system.^35^

### Implementation of the Intervention

**FIGURE 3:**
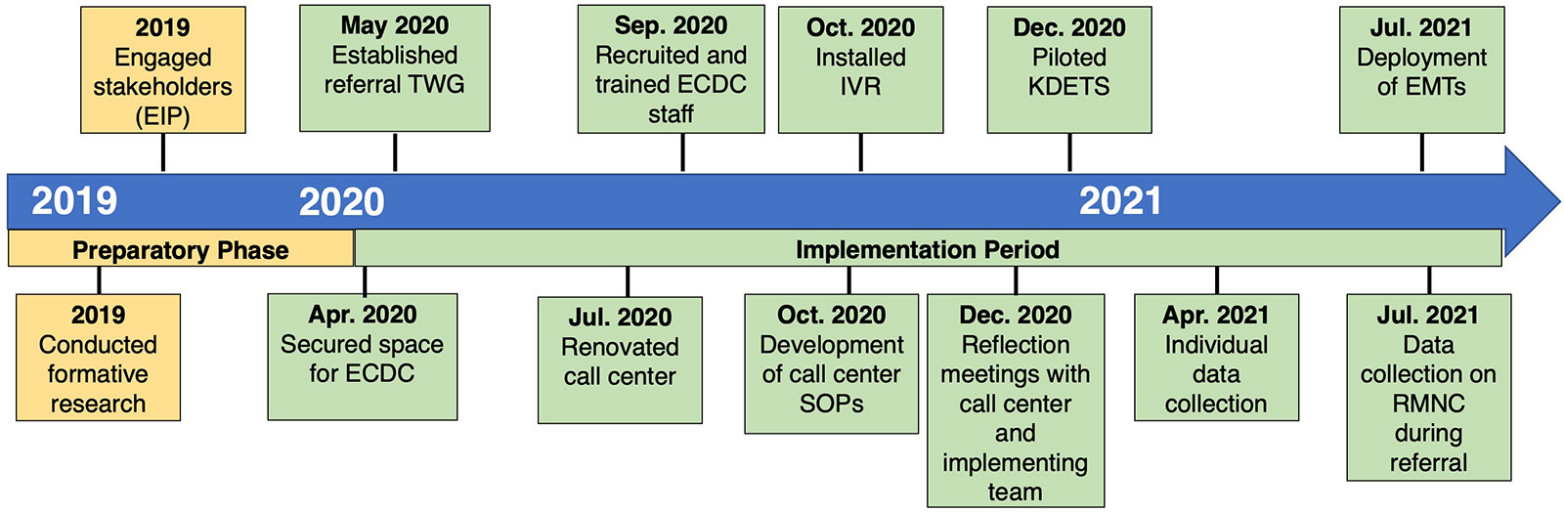
Implementation Timeline for the MaNe Project Activities Abbreviations: ECDC, emergency call and dispatch center; EIP, empathy insight prototyping; EMT, emergency medical technician; IVR, interactive voice recorder; KDETS, Kampala Digital Emergency Transport System; RMNC, respectful maternal and newborn care; SOP, standard operating procedure; TWG, technical working group.

We describe the activities undertaken as part of MaNe’s implementation research to establish a functional ECDC in Kampala. Figure 3 shows the timelines of different MaNe activities, while Table 1 describes the details of the different activities.

**TABLE 1.** Steps in MaNe Intervention Phase in Establishing Emergency Call and Dispatch Center in Uganda

**Steps**	**Description of Activities**
Equip the ECDC and ambulances	April 2020 Secured space for the ECDC within KCCA Headquarters at City Hall.
	July 2020 Renovated the center and equipped it with basic equipment, including computers, phones, headsets, ambulance tracking screens, furniture, and Internet connectivity. MaNe provided seed funding for renovating and equipping the center. KCCA provided 6 ambulances that were accessed through the call center. KCCA supervised the operation of ECDC.
Form an advisory TWG for the referralsystem	May 2020 Formed referral TWG to provide technical input in the ECDC establishment and operation. Referral TWG membership included representatives from the MOH, KCCA leadership, implementing partners (Malteser International, Emergency Care Society Uganda), professional bodies and associations, and emergency medical service training institutions. The TWG and MaNe team held virtual monthly meetings to discuss referral gaps, including those related to the COVID-19 lockdown.
Recruit and train call center agents	September 2020 Recruited and trained 9 call and dispatch staff as call response officers and shift supervisors. Held a 2-day training based on the existing MOH national call and dispatch manual. Content had roles and responsibilities of the call center staff, skills in answering calls and advice on maternal and newborn referrals, dispatch of ambulances to appropriate referral facilities, and how to make follow-ups for referral outcomes. The center functioned 24 hours/day and ran in 3 shifts.
Develop referral standard operatingprocedures and checklists	October 2020 Developed referral standard operating procedures, which had guidelines on pre-referral stabilization of cases, stabilization during referral, respectful maternal and newborn care during referral, and transportation of the mother and/or the newborn. MaNe staff and the MOH inspected the ambulances regularly to ensure quality and safe transport during patient transfer. To perform the inspection, the project adopted a tool used by the emergency medical services department of the MOH for licensing ambulances and ambulance service providers.
Engage MOH officials and other stakeholders	The establishment of the ECDC in April 2020 coincided with the onset of the COVID-19 epidemic in Uganda. The COVID-19 technical team from the MOH supported the training of the call center agents and ambulance drivers on COVID-19 response and care of people with COVID-19 during referrals. MOH provided guidelines on responding to queries and concerns by the public related to COVID-19. During the second wave of COVID-19 that started in May 2021, the MOH, Uganda Red Cross, and KCCA formed a joint resurgency team to coordinate evacuation of patients during the COVID-19 lockdown through the ECDC. The MOH and Uganda Red Cross provided additional 8 and 5 ambulances, respectively, together with emergency medical technicians specifically for suspected COVID-19 cases. Five KCCA ambulances were reserved for referrals not related to COVID-19 (mainly maternal and newborn cases), and their deployment was coordinated at the ECDC.
Generate awareness about ECDC	The MaNe project generated provider and community awareness about the call center through outreach at public and private health facilities. Distributed the ECDC toll-free number to pregnant women. Displayed wall stickers with the ECDC toll-free number at health facilities in locations where they could easily be seen by community members. Linked interested public and private facilities to the ECDC. Promoted information about the ECDC through KCCA social platforms, like Twitter, Instagram, and WhatsApp.
Train facility providers, ambulance drivers,and ECDC agents on KDETS	December 2020 Piloted the KDETS application with 4 public facilities and 4 private facilities. Oriented health providers and ambulance drivers on the use of the KDETS application, including how to download and access the application and enter the information. Made available continuing support to the participating health facilities to assist in the adoption of the application. Provided a telephone hotline to assist with any application or service-related problems. Provided facilities and drivers with tablets for pilot implementation of the KDETS application. The project team monitored the uptake of the application, its benefits, and practical challenges faced by health care workers (e.g., Internet connectivity, concerns about additional work, and logistics). Drawing on learnings from the pilot, scaled up application training to 72 facilities and 25 ambulances.

Abbreviations: ECDC, emergency call and dispatch center; KCCA, Kampala Capital City Authority; KDETS, Kampala Digital Emergency Transport System; MaNe; Kampala Slum Maternal Newborn project; MOH, Ministry of Health; TWG, technical working group.

### Data Collection, Management, and Analysis

A mixed-methods study design was used consisting of quantitative data analysis of demand for the ECDC using call center logs and qualitative in-depth interviews (IDIs) to document facilitators and barriers to the implementation process. A data collection matrix tool was created to capture all call requests to the ECDC. Data from April 2020, when implementation began, through June 2021 were included in the analysis. The Microsoft Excel-based tool included fields for relevant biographical and demographic information, reasons for the call, and referring and receiving facilities. The ECDC data were reviewed on a weekly basis. Data were cleaned in Microsoft Excel. With guidance from an obstetrician on the research team, the reasons for calling were classified into categories of maternal and newborn conditions. Data were exported to Stata 12 (StataCorp, College Station, TX) for descriptive analyses.

The qualitative data collection was conducted in April 2021 to answer research questions about the feasibility, acceptability, and sustainability of the ECDC system and mobile application. Twenty-six IDIs were conducted using a semistructured interview guide. Interviews were conducted at venues convenient to the participants by 2 researchers experienced in qualitative research. Participants were health providers and proprietors purposively selected from the 4 public and 5 private facilities that had received training and referred patients through the call center. In addition, the MaNe research team interviewed ambulance drivers, KCCA officials, and women who had utilized referrals using the ambulances (Table 2). To reduce redundancy and minimize respondent burden, the sample size was established based on familiarity and saturation. Participants were compensated for their time with Ugandan shillings that was equivalent to US$6.

**TABLE 2.** In-depth Interview Participants for the Kampala Referral System Intervention

**Institution**	**Respondent**	**No.**
Private facilities	Nurse/midwives	6
	Clinical officers (proprietor)	1
	Doctor (proprietor)	2
Public facilities	Midwives	8
Kampala Capital City Authority ambulance	Ambulance drivers	2
Kampala Capital City Authority representative	District medical officer	2
Community representative who used referral ambulance	Referred women	5
	**Total**	26

The interviews were audio-recorded and transcribed verbatim. Analysis was conducted by 2 researchers with experience in qualitative research. The analysis comprised both inductive and deductive approaches guided by the consensual qualitative research procedure to understand inner experiences, attitudes, and convictions with the use of the referral system.^36^ Two coders conducted line-by-line coding of the transcripts using Atlas.ti (version 9). Codes were applied based on emerging themes and the project objectives. After coding, the research team convened to compare the coded data to ensure consistency and reliability. Overarching themes were identified from the coded transcripts, and quotes representing the themes were extracted.

In addition to the IDIs, the implementing team conducted periodic pause-and-reflect discussions during the implementation of the program to explore what was working well and why, to make any changes, and to document key activities, challenges, and adaptations occurring over the course of implementation. To guide these discussions, the team used a structured template with the following sections: (1) objective and activity being implemented; (2) relevant updates on the implementation process; (3) successes, including what worked well and why; (4) challenges, including what did not work well and why; (5) what has changed or stopped; and (6) what should be done next. The team guided these discussions, which lasted approximately 60–90 minutes, with KCCA officials, ECDC agents, health providers, ambulance drivers, and members of the mobile application technical team or developers. Reflections were facilitated by a member of the research and knowledge management team and supported by a notetaker to capture detailed discussion notes.

### Ethical Approval

Approval for this research was provided by the Mildmay Research and Ethics Committee (REC Ref 0508-2020) and the Uganda National Council for Science and Technology (HS962ES**)**. Study participants provided their written informed consent.

## RESULTS

### ECDC Calls and Ambulance Transport

**FIGURE 4:**
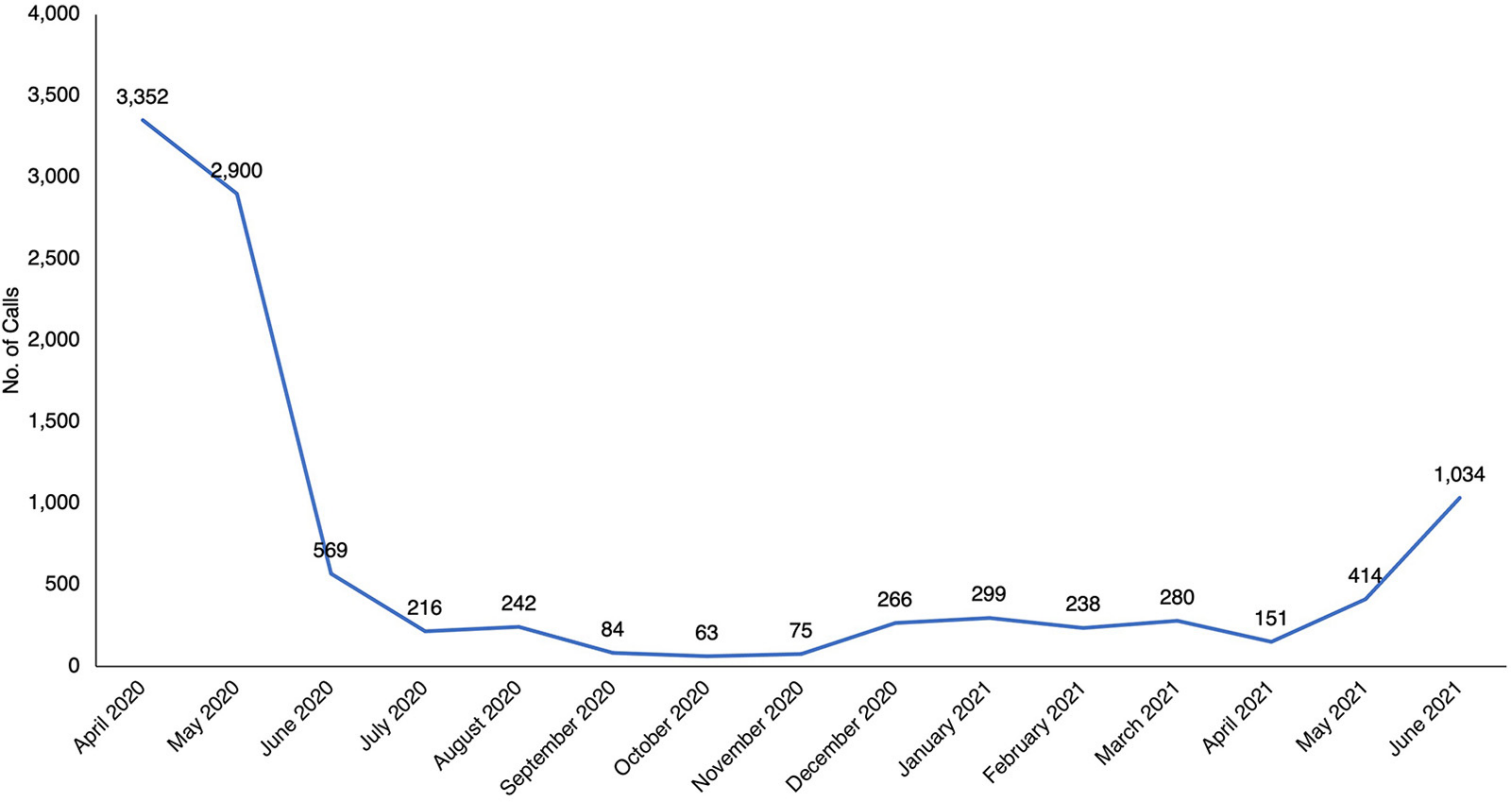
Monthly Calls to the Kampala City Emergency Call and Dispatch Center, April 2020 to June 2021

Between April 2020 and June 2021, a total of 10,183 calls were made to the ECDC. Sixty-one percent of these calls were recorded in the first 2 months of the establishment of the ECDC (April and May 2020), which corresponded with the first COVID-19 lockdown in Uganda. As shown in Figure 4, calls increased in June 2021, which corresponded to the second lockdown instituted by the Government of Uganda to control the second wave of COVID-19 cases.

**FIGURE 5:**
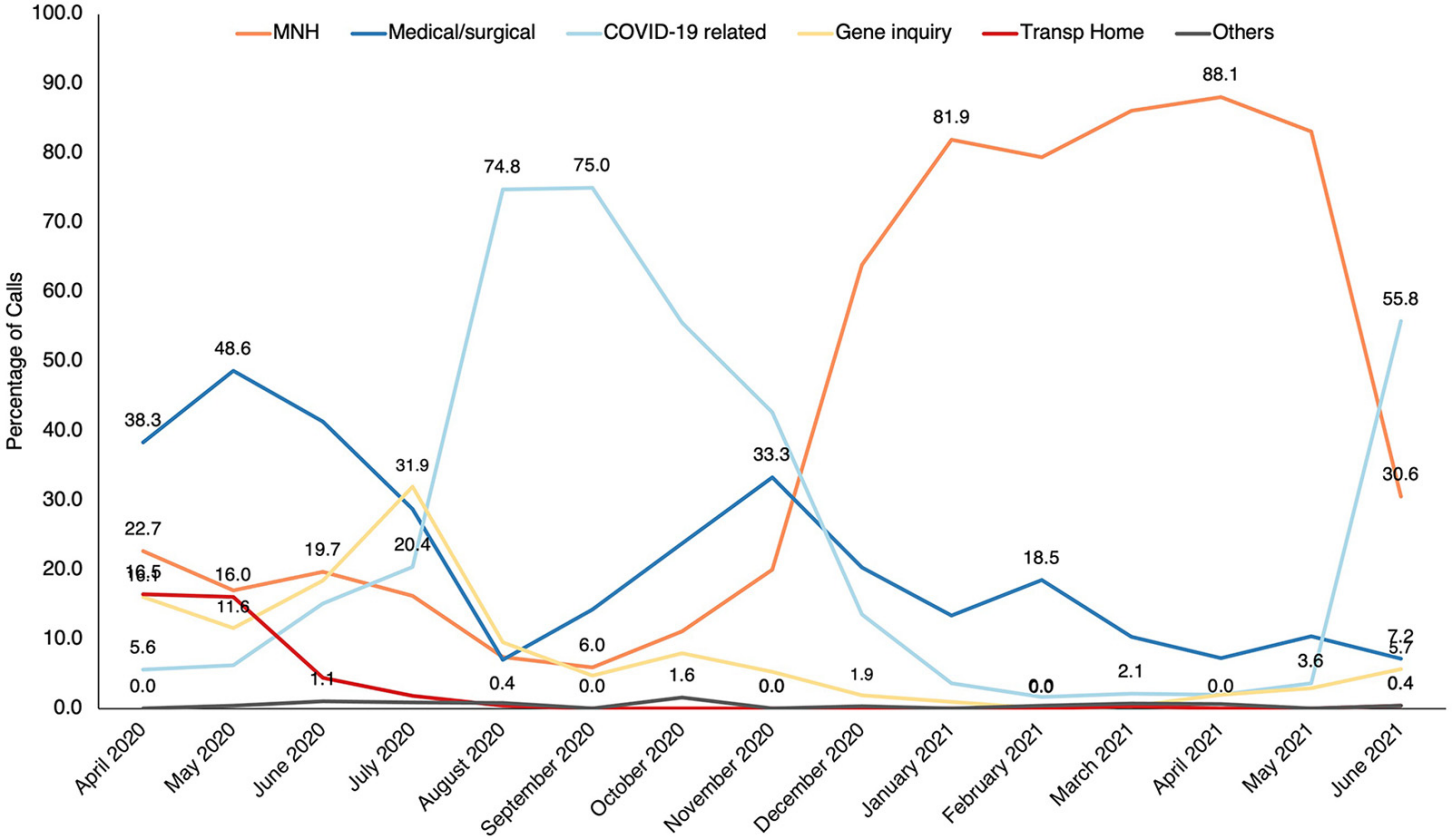
Reasons for Calls to Kampala City Emergency Call and Dispatch Center, April 2020 to June 2021 Abbrevations: Gene, general; MNH, maternal and newborn health; transp, transport.

As shown in Table 3, 25% (2,583) of calls were for maternal health and 3% (328) were for newborn health issues. Of the 2,911 MNH calls, 84% (2,446) of the cases received ambulance transport (referred) through the center. Though the ECDC was established specifically for MNH requests for ambulance transport, the service was not restricted to these cases only during the COVID-19 pandemic. One-third of calls to the ECDC were for medical or surgical cases requesting transport for an emergency medical condition, retrieval of antiretroviral medication from clinics, scheduled cancer therapy, a surgical or accident problem, transport for a mental problem, or for routine care of preexisting chronic diseases, like diabetes and hypertension. Calls related to COVID-19 (14%; 1,463) were people reporting a suspected case in the community or health facility requesting transport for evacuation or referral. In addition, 12% (1,169) of calls were general inquiries about restrictions during the lockdown, where to go for COVID-19 vaccinations, relief support, or KCCA administrative guidance. About 10% (1,053) of calls were requests for transport back home after medical treatment. Transport back home was requested mainly during the first lockdown (April 2020 to June 2020) (Figure 5).

**TABLE 3.** Reasons for Calls to the Kampala Emergency Call and Dispatch Center, April 2020–June 2021

**Call Categories**	**No. (%)**
Maternal health-related calls(n=2,583, 25.4%)	
Pregnancy or postnatal check-up	547 (5.4)
Mother in labor	471 (4.6)
Prolonged/obstructed labor	424 (4.2)
Hypertensive disease in pregnancy	223 (2.2)
Obstetric hemorrhage	205 (2.0)
Preterm labor	159 (1.6)
Fetal distress	142 (1.4)
Previous cesarean delivery scar	112 (1.1)
Spontaneous abortion	90 (0.9)
Premature rupture of membranes	54 (0.5)
Intrauterine fetal death	30 (0.3)
Other pregnancy complications	126 (1.2)
Newborn health-related calls (n=328, 3.3%)	
Birth asphyxia	152 (1.5)
Neonatal infections	77 (0.8)
Premature baby	81 (0.8)
Birth defect	18 (0.2)
Child illness	173 (1.7)
Medical/surgical related calls(n=3,356, 33.0%)^a^	
Medical emergencies	1,259 (12.4)
Routine care	1,224 (12.0)
Antiretroviral drug refills	417 (4.1)
Cancer	270 (2.7)
Mental health	107 (1.1)
Road traffic accident/surgical	79 (0.8)
General inquiries	1,169 (11.5)
Transport home	1,053 (10.3)
Abuse	14 (0.1)
COVID-19 related	1,463 (14.4)
Dead body	34 (0.3)
Gender-based violence	10 (0.1)
**Total**	10,183 (100.0)

^a^Though the pilot was initially meant for maternal and newborn health referrals, due to COVID-19 movement restrictions, the communities were assisted in seeking care and transport to the facility through the emergency call and dispatch center.

Though the ECDC was established specifically for MNH requests for ambulance transport, the service was not restricted to these cases only.

**FIGURE 6:**
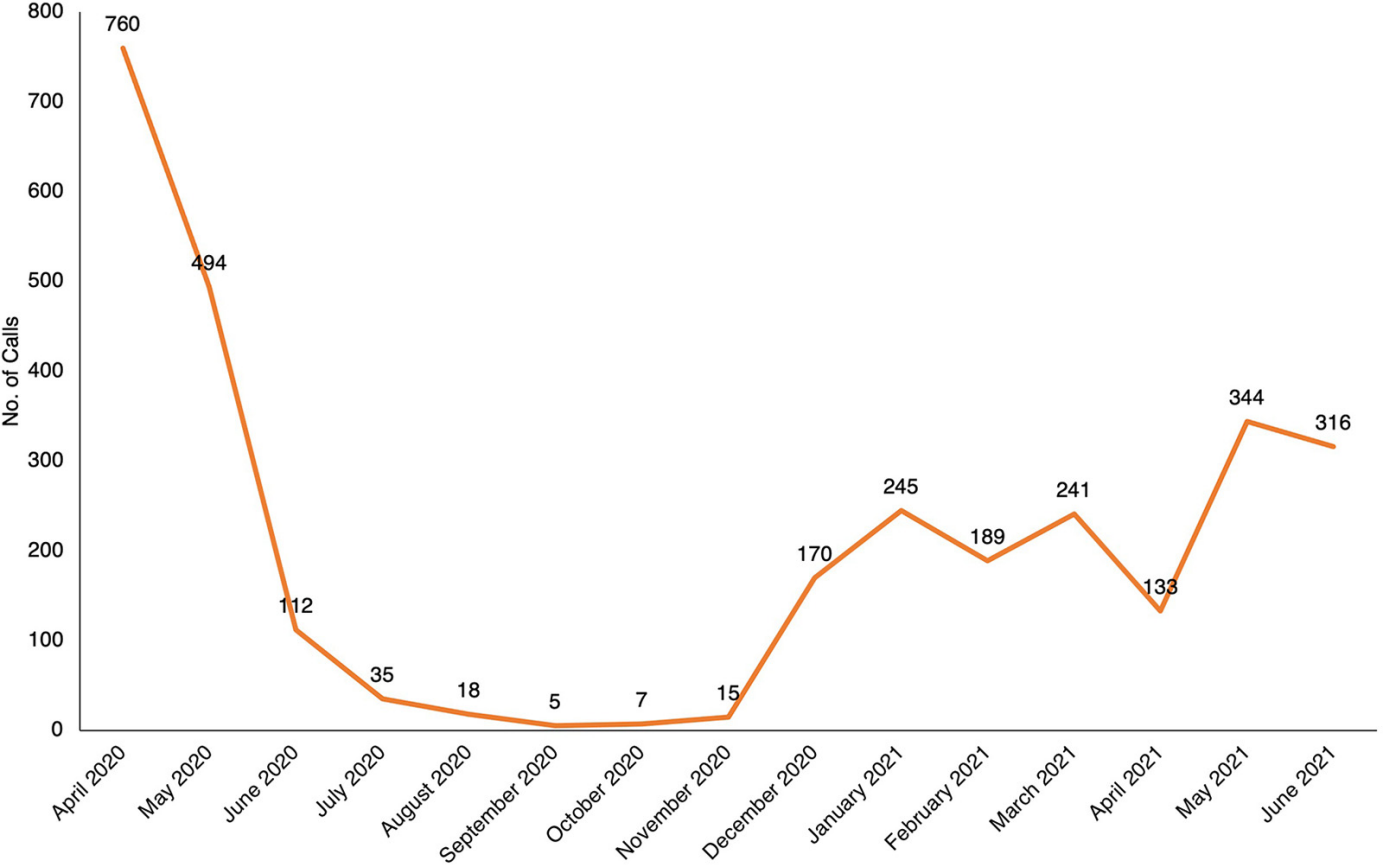
Trend of Maternal and Newborn Health Calls to the Kampala City Emergency Call and Dispatch Center, April 2020 to June 2021

Figure 5 shows the reasons for the calls to the ECDC over time. As observed, in the second half of implementation (October 2020 to May 2021), most calls were for MNH until the second wave of COVID-19 (May 2021 to June 2021). Figure 6 shows the total MNH calls from April 2020 to June 2021.

**FIGURE 7:**
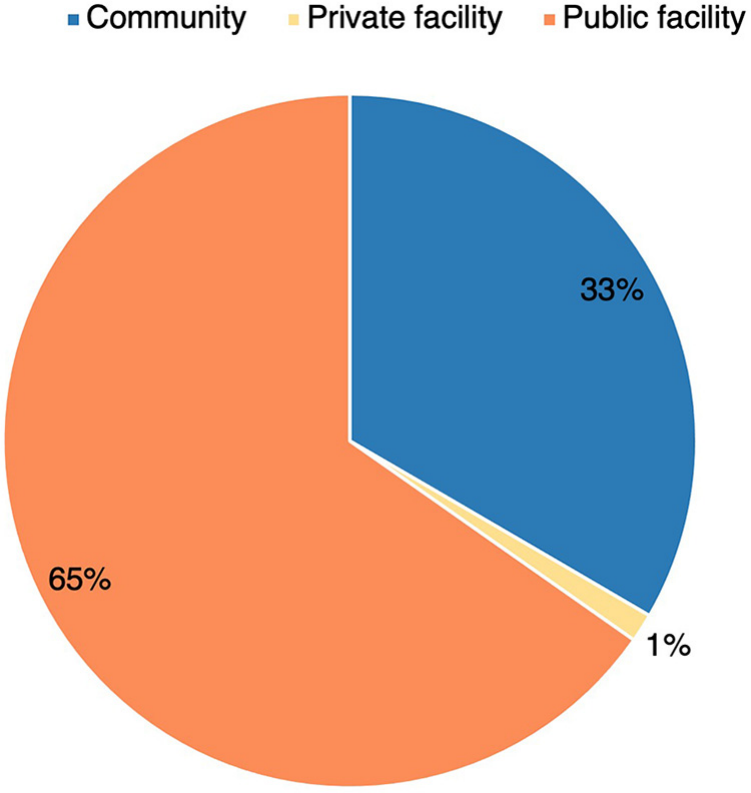
Origin of the Maternal and Newborn Health Calls to Kampala City Emergency Call and Dispatch Center, April 2020 to June 2021

Of the 10,183 general calls to the ECDC, 63% (6,404) were from the community, while 32% (3,227) and 5% (552) were from public and private facilities, respectively. In contrast, of the 2,911 MNH calls, 65% were from public health facilities, followed by community (33%) and private facilities (1%) (Figure 7). The common reasons for community MNH calls to the public and private ambulances were requests for transport for a mother in labor, to attend antenatal care, bleeding in pregnancy (including spontaneous abortion), other pregnancy complications, sick newborn/child, or premature baby.

Table 4 shows the different actions that were taken by the ECDC staff in response to a call. In 35% (3,595) of cases, an ambulance was dispatched to transfer or evacuate a patient. KCCA ambulances were specifically dispatched to transport obstetric and newborn emergencies, while MOH ambulances transported COVID-19 suspected or diagnosed cases. KCCA vehicles were also available to respond to calls for transport for antiretroviral drugs, routine medical appointments, and clinic reviews. The KCCA vehicles were double-cabin pickups designed not to carry critically ill patients. For 29.7% of the calls, the ECDC staff provided information on where to seek COVID-19 screening, where to obtain a travel permit during lockdown, how to handle a neighbor who is sick, and where to obtain relief food during the COVID-19 epidemic.

**TABLE 4.** Actions Taken by Call Center Staff in Response to Calls

	**No. (%)**
Contacted KCCA ambulance team	3,595 (35.3)
Contacted Ministry of Health ambulance	367 (3.6)
Linked to district health officer	120 (1.2)
Linked to KCCA vehicle	1,860 (18.3)
Caller linked to ground surveillance team	352 (3.5)
Counseled	333 (3.3)
Linked to other KCCA official	526 (5.2)
Other actions (e.g., provided information)	3,030 (29.7)
**Total**	10,183 (100.0)

Abbreviation: KCCA, Kampala Capital City Authority.

### Acceptability of Emergency Call and Dispatch Center

Most participants interviewed acknowledged the ECDC as an acceptable intervention. The following themes emerged: efficient communication and information flow between health facilities, improved referral services, and equitable duty allocation and reduced workload.

#### Efficient Communication and Coordination

According to most participants, the establishment of the ECDC improved communication and information flow between health facilities, the call center, and ambulance drivers. Interviewees expressed appreciation for the ECDC’s coordinating role and the support it provided in identifying an ambulance and referral facility to best handle the medical condition. The ECDC also provided health centers with information on which driver was picking up the patient, which made it easier for facility staff to contact the ambulance if needed. In addition, the call center informed the health facility where the patient was supposed to be referred and notified the referral facility, which allowed them to prepare in advance.

*The call center is good because previously before it came, we used to have issues. We used to call these ambulance drivers directly, and you could get scenarios where you call one and he tells you he is not able, call “so and so.” When you call the next, they even don’t pick [up]calls. We had those issues but now, the center has even eased our work in that we call, and it is up to them. They get an ambulance driver, and they inform us of who is coming, which is very good.* —Health care provider, public health facility

Interviewees expressed appreciation for the ECDC’s coordinating role and the support it provided in identifying an ambulance and referral facility to best handle the patient’s medical condition.

Participants also reported that communication and information flow through the ECDC improved coordination of referrals. Through this system, the referral or receiving facility was informed of the condition of the patient being referred. The patient was given priority on arrival, which was not the case before.

*When we call the call center, the facility we are referring to, gets to know that this patient is from Victoria Maternity Care, and she needs such and such assistance. They are prepared to receive the case. I think it helps the patients get priority especially with complex health cases and in a way, it also eases on our work.* —Health care provider, private health facility

A medical officer from the KCCA discussed how the ECDC improved efficiency and the ability to provide more timely care.

*Referring a mother without notifying the receiving facilities is a mistake because it breaks continuity of care. Therefore, notifying the receiving facilities is important in ensuring timely care. This facilitates the receiving facility to prepare and prioritize for the incoming emergency.* —District medical officer, KCCA

Acceptability was facilitated by the availability of the ECDC toll-free number, which was reported to be fast compared to other toll-free numbers in the city. Participants also acknowledged that whenever they made a phone call, it would go through without any difficulties. Provision of tablets and airtime to the facilities and drivers helped improve communication.

*In various occasions the connection is not bad, for example calling ECDC is better than calling police 999 center …[laughs]. The call center is faster.* —Ambulance driver

*Yes, they have given us a card. Every month we have a handset we use to call… The call center is toll free… We have a phone for the facility.* —Health care provider, public health facility

#### Improved Referral Services

Study participants mentioned that the ECDC dispatched ambulances on time, whereas before, they struggled to find one. This made the referral process faster. In addition, patients did not pay for the service rendered. Furthermore, patients who were transported using those ambulances were given priority in the receiving health facilities, shortening the arrival to intervention time, which in turn, affected the patients’ outcomes.

*The impact the center has made is that it is quick, quick transportation and when a mother is taken by the ambulance, it is free, and they are given priority where they have been referred. They work on them very first. So, I would say that the impact is good.* —Health care provider, private health facility

In addition, participants reported that the application enabled referral facilities to prepare for incoming referred patients because the details of the patient had been sent in advance to the receiving facility. Some participants also mentioned that the application was useful for communicating with receiving facilities about incoming emergencies, which enabled medical workers to prepare for incoming emergency cases. Health providers also reflected that the early notification of emergency cases by the application allowed them to prepare emotionally in addition to preparing the supplies.

*The good thing with the application is that by the time the patient reaches referral facility, they already know the emergency coming and they are prepared.* —Provider, public facility

Most participants reported a reduction in delays in obtaining fuel and during transit. The ambulance arrived at the referring facility already fueled. Previously, health workers in the referring facility would seek authorization for a fuel voucher for the ambulance when the budget line was not exhausted. Currently, the fuel budget for running the ambulances is controlled by the KCCA, which has a much larger allocation compared to the previous facility budget line.

#### Equitable Duty Allocation and Reduced Workload

Before the establishment of the ECDC, few public facilities had a designated ambulance and driver. Each facility had a different number of referrals, and ambulance drivers’ duties and workloads varied. Ambulance drivers were pleased that the ECDC helped in coordinating and assigning duties and distributing workloads evenly among drivers. Duty coverage improved, and in situations where a driver was absent or when the ambulance was broken down, there was reassurance of continued referral service from the pool of ambulances and drivers.

*To me they are doing a good job. Even when you call those people at the facilities, it is working for them. The call center is working for all of us. Just imagine before the call center there was a driver attached to a facility. If the facility got many cases and they get an urgent case when the ambulance is in transit you know what it means…. They are very efficient, if they called me and I am having a bath and I don’t pick my call, they get someone else, but before when I was attached to a facility, if I don’t pick my calls they have to wait and yet the cases are very urgent like PPH [postpartum hemorrhage]. The coordination at the call center is perfect.* —KCCA ambulance driver

Ambulance drivers were pleased that the ECDC helped in coordinating and assigning duties to distribute workloads evenly among drivers.

Health providers reported that the ECDC reduced the burden of arranging for referral transport and the challenge of locating an ambulance driver. Providers were able to concentrate on providing care rather than arranging transport.

*Before the establishment of call center, we used to call these ambulance drivers directly and he assures you he is not available. The call center has eased our work in that we call, it is up to them to get the driver, and they just inform us of who is coming, which is very good. We are now able to care for the women.* —Health care provider, public health facility

### Feasibility of Establishing the Call and Dispatch Center

The following themes related to the feasibility of establishing an ECDC emerged from the interviews and from the review of the meeting minutes: supportive leadership of KCCA, training the call center team, and equipping the center and ambulances.

#### Supportive Leadership of KCCA

The project’s collaboration with the district leadership facilitated the establishment of the ECDC. The district’s leadership was central in providing office space, a telephone line, and Internet access to the ECDC. The Director of Public Health and Environment had a central role in decision-making and facilitated the use of evidence from the implementation research. He actively participated in local and international dissemination of the research findings.

#### Training of Health Providers, Ambulance Drivers, and ECDC Agents

Training was based on the existing MOH national call and dispatch manual. The health providers, call center agents, and drivers were trained on the function of the ECDC, roles and responsibilities of call center staff, how to answer calls, advice on MNH referrals, dispatch of ambulances to appropriate referral facilities, and how to make follow-ups for referral outcomes. The telephone number to refer a client was provided to each agent as well.

*For the call center, there is a number we were given when I went for that training that was at Hotel Africana, that in case you have an emergency or if you want to refer a mother or a baby, you call that number and they send you an ambulance, any government or KCCA ambulance or if not, in case these ones are busy they can send you any private ambulance free of charge but it is KCCA to pay for it.* —Health care provider, private facility

#### Equipping the Center and Ambulances

Participants mentioned that it was easy to connect to the call center. Although ambulances were in good mechanical condition, participants commented that they lacked basic equipment, prompting them to ask the KCCA to equip the ambulances.

Participants mentioned that there was effective communication and information flow among the stakeholders (e.g., call center agents, drivers, and health workers). However, because call center agents were not health workers, their understanding and triaging of health inquiries posed a challenge, so participants recommended recruiting health workers to staff the call and dispatch center to address this challenge.

#### Cost for Establishment of ECDC

With seed funding from USAID, approximately US$263,000 was invested in the establishment of the ECDC. The bulk of the funds (approximately 60%) was on fixed costs that included refurbishment of the call center space; equipment, computers and accessories; and mobile application development. The remaining 40% of the funds were running costs that included staff salary, training, transport, Internet, and food. When the USAID-funded MaNe project closed, the directorate of public health at KCCA took over the call center operating costs.

### Acceptability of the Mobile Application

The following themes on acceptability of the mobile application emerged from the qualitative interviews: easy to use and improved accountability.

#### Easy to Use

Both ambulance drivers and most health providers reported that the application was reliable and very suitable for referral purposes. They stated that the mobile application was easy to use, provided sufficient storage for patient information, and allowed for adequate follow-up of referrals because all the details of the referral process were stored in the application.

*Me, I don’t see any problem with it; it is good; it is easy. —*Health care provider, public facility

*It is very easy to use; you just swipe, when you get an alert accepting, you just swipe. When you need something, you swipe too. —*Ambulance driver

#### Improved Accountability

The findings show that the ECDC has improved the coordination and accountability of the ambulance team. The mobile application allows for monitoring the patient’s journey and confirming that the patient has reached the receiving facility. Participants reported that the ambulances’ response time improved because of monitoring at the ECDC.

Participants reported that ambulances’ response time improved because of monitoring at the ECDC.

### Sustainability of the ECDC

The district leadership has taken on the operating costs of the center, including staff salary, Internet connectivity, and ambulance fuel. Meanwhile, at the time of this writing, plans were being developed to introduce a total market approach where more well-off urban residents and users of the ambulance with health insurance could be levied a fee to use the ambulance and maintain it free for the urban poor. In addition, MaNe engaged an emergency medical technician (EMT) training institution, Lubaga Hospital, to explore the possibility of their students doing internships at the KCCA call center and in the ambulances under the supervision of the EMS Coordinator. This was aimed at cost reduction for call center human resources. A memorandum of understanding was drafted by the training school and was under review by the KCCA legal team by the close of the MaNe project. When the memorandum has been signed, the student EMTs will be deployed and trained on the operations of the call and dispatch center as well as the ambulances under the oversight of the EMS steering committee.

KEDTS was integrated into the DHIS2 testing environment with support from the DHIS2 developers and the KCCA biostatistician. This was after the development of the relevant MNH variables and their indicators. Partners have been mobilized to finalize discussions with the MOH to implement the KEDTS-DHIS2 linkage. Integration of the KEDTS system to DHIS2 will facilitate access to the referral cases and guide decision-making at the MOH and different levels of management. USAID partners have been mobilized to pursue the completion of this linkage process through the necessary engagements with the MOH.

The project team disseminated the implementation outcomes of ECDC at national conferences and meetings with MOH stakeholders. There was very strong interest from the MOH, local district governments, and partners to replicate the interventions countrywide. However, learnings show that time and resources for strengthening provider knowledge, skills, and confidence in the use of the new technology were necessary for the smooth uptake of the KDETs. The use of a dedicated short code or toll-free line seems more acceptable to users and stakeholders.

### Challenges to Establishing the ECDC and Implementing the Mobile Application

The following themes emerged as challenges to establishing the ECDC and implementing the mobile application: few and inadequately equipped ambulances, unmet expectations of ambulance drivers, cost of data and poor Internet connectivity, and providers who were not tech-savvy.

Ambulance drivers expressed that the ambulances were few, and some of them lacked the necessary amenities like oxygen, stretchers, gloves, cotton, and beds. In addition, some ambulance sirens were faulty, which made navigating traffic jams difficult and ultimately contributed to delays in arrival. Although the leadership of KCCA had budgeted for equipping ambulances, there were bureaucratic delays in the procurement process.

Ambulance drivers expressed that the number of ambulances were few and some of them lacked the necessary amenities.

Public ambulance drivers (KCCA) initially perceived the new ambulance system as “project work” or additional work that deserved compensation. They reported that since the establishment of the ECDC, they worked overtime, including weekends and public holidays. When they compared themselves with private ambulance drivers, the latter were given an allowance by their companies in addition to their salary. This resulted in a lack of enthusiasm among the public ambulance drivers.

*The passion I have on the MaNe project will be different from my colleagues; some do not want to hear anything related to the MaNe project, because they are not earning anything from it. The drivers and health providers at facility are the foot soldiers and the implementers of this project. They are not motivated, because the MaNe project is not offering any allowance to them.* —Ambulance driver

Participants stated that insufficient Internet data hindered the operation and use of the application. The Internet bundle provided was not enough, and participants suggested that more funding for Internet data should be allocated for the operation.

*KCCA should first sit and understand what they are doing. They plan how to manage the system; how can it have no data that is reliable and consistent? I receive 1GB of data per month, which cannot be enough for me. I think these phones should have unlimited Internet connection.* —Ambulance driver

Some facilities or locations faced poor network connectivity or unstable Internet, which sometimes caused the system to freeze. In addition, not all providers and drivers used the system, and some were reluctant to use the application, which had a negative impact on other interested colleagues. In reflection meetings, some health workers stated they were not comfortable using the application due to the more advanced knowledge of technology required to use it. Some encountered a challenge inputting data and were unable to provide enough information for emergencies. According to a few participants, it was difficult to operate the application, such that some providers had to first call the ECDC to get help on how to input information. One private proprietor stated:

*It was quick and effective but maybe the other challenge that it had was the network. However, it sometimes failed to upload the information. So, I would first call the ECDC to secure the ambulance and later feed in this information because it was so disturbing.* —Proprietor, private health facility

Providers stated that there were time constraints whereby a health worker who was busy attending an emergency case would not have time to put the needed information in the application before the referral was made.

*But sometimes when you are busy you fail. When you are busy, I request a colleague to send the message as I prepare the mother or [I] send the message after the referral. —*Health care provider, public facility

## DISCUSSION

In this implementation research study, the MaNe team assessed the feasibility, acceptability, and sustainability of establishing an ECDC and deploying a mobile application to track ambulance drivers in Kampala. Overall, most providers said that the ECDC and the application enabled efficient communication, improved referral services and equitable duty allocation, and reduced workload. These positive findings were likely due to the fact that the stakeholders saw the interventions as practical and realistic for increasing access to care by addressing gaps in the referral pathway. Factors that were facilitators to feasibility, acceptability, and sustainability, including support by KCAA leadership, efficient communication and coordination, and improved equitable duty allocation and accountability. A few participants reported challenges with poor Internet connectivity and the cost of the Internet bundle. Currently, the call center is housed in government space, using government IT structures. KCCA was involved in developing the application and thus can continue with it even when the MaNe project ends, with the government paying some operational costs.

Most providers said that the ECDC and the application enabled efficient communication, improved referral services and equitable duty allocation, and reduced workload.

The original plan for the MaNe project was to improve the referral of maternal and newborn care emergencies. However, due to COVID-19, the ECDC became the only available coordination center for referrals for all emergencies. In fact, our data show that the majority (60%) of the calls were recorded in the first 2 months of establishment of the ECDC. This period corresponded with the first COVID-19 total lockdown, where movement was restricted to only essential workers. The majority of people were not prepared for the lockdown, and the call center questions reflected a wide variety of areas, such as inquiring about food rations promised by the government, asking about COVID-19 management and restrictions, learning where to get movement permits, reporting community member violations of COVID-19 lockdown guidelines, transporting essential workers to duty and back home, asking about the availability of treatment, as well as other questions. In addition to serving as a referral coordination point, the call center was an important source of information for the community and health providers. The decline in the call volume reflects the period when the government lifted the COVID-19 lockdown and a sense of some normalcy returned in the city. The ability of the ECDC to accommodate COVID-19 pressure for referral coordination highlights the flexibility of the intervention in settings with limited resources and to provide life-saving services. It shows that, in resource-limited settings, having a referral system for only MNH services is not cost effective.

Involving key stakeholders, such as MOH members, district leadership, health providers, and community leaders, in the design and planning phases promoted the acceptability and practical implementation of the intervention. Meaningful engagement of the district director of Public Health and Environment as a co-investigator of the research likely influenced the acceptability and feasibility of implementation. His office held a degree of authority and influenced wider decisions on implementation. His participation in the dissemination of results promoted acceptability and ownership of research findings. The value of collaborating with decision-makers and policymakers in implementation research has been cited in the literature as a pivotal approach to promoting the uptake of research findings and, eventually, the sustainability of programs.^37^^–^^39^ The MaNe project experience corroborates this evidence. Involvement of policymakers in implementation has also been shown to reduce research waste in terms of time and effort spent on producing irrelevant or unusable evidence.^38^^,^^40^

There is a gap in the evidence base in low-income settings on the role of a call and dispatch center for improving the communication and coordination of ambulances for referrals. A study in Indonesia and another from rural Ghana launched transportation and communication systems to mitigate barriers associated with access to care. These studies showed that community endorsement of the initiative increased emergency referrals to higher-level facilities; improved management of obstetric and newborn complications, including stabilization of cases before referral; and improved preparedness of the referral facility.^41^^,^^42^ Most of the literature originating from low-income countries, like Uganda, stress the importance of communication but lack the coordination component that a call and dispatch center provides.^12^^,^^19^ Maternal health experts have stressed that communication efforts that are integrated with transport and improved service delivery efforts may help reduce mortality and morbidity.^43^

In the MaNe project, having a toll-free number at the ECDC and giving health care providers and drivers mobile phone airtime and tablets dedicated to referrals facilitated communication and coordination efficiency. Provision of airtime and data beyond the MaNe project may be challenging, affecting the sustainability of the project. The MaNe project engaged the KCCA leadership on this point, and KCCA agreed to allocate funds to airtime and tablets in their budget. The current KCCA annual health budget is approximately US$8.7 million. Of this, 3.1% is allocated for the city referral system, specifically, salaries for 18 call center agents and 12 drivers, office maintenance, insurance, fuel, and repairs for a fleet of 12 ambulances. However, we do not know how long this funding will be sustained. From the literature, both the funders, community providers, and researchers recognize the importance of program institutionalization after closure of a health project. Studying its sustainability is challenging.^44^ There is a potential loss of investments and people after the closure of the program. In addition, some of the behavioral changes are only detectable 3–10 years (latency period) after the beginning of the project.^45^ We acknowledge that the KCCA budget may not match the amount of funding during the MaNe project implementation. However, there has been political support for program adaptation and appropriation of the budget for more staff and additional ambulances.

Furthermore, a partnership with private training EMT institutions has provided trainees and private ambulance owners, and engagement with a USAID-funded Maternal Child Health and Nutrition project has provided vital support for the continuation of the referral system. We acknowledge that the MaNe project did not cover all the constructs in a 9-domain conceptual framework for program sustainability framework by Schell et al.^46^ Nevertheless, the political will, partnerships created, and engagement of other implementing partners are more likely to facilitate the continuation of the referral system. However, continued assessment after the latency period using an established framework is important to provide lessons for sustainability and scalability when funding is minimal.

Political will, partnerships created, and engagement of other implementing partners are more likely to facilitate the continuation of the referral system.

With smartphones becoming the technology of choice in the country, the acceptability of the mobile application is promising for continued use and uptake. The application’s added advantage of relaying patient information to the receiving facility in advance allowed health workers time to prepare for the incoming emergency.

The findings presented here also indicated that the ECDC promoted the equitable distribution of duties among ambulance drivers. In settings like Uganda, where there is a shortage of human resources for health, it is common to find that some cadres are more overworked than others. This causes burnout and a tendency to abscond from duties. Before the intervention, ambulance drivers were attached to specific health facilities, but now there is 1 command center, and drivers respond to all facilities and communities. With the application, ambulance movements are monitored through GPS, which has made drivers more accountable for responding to an emergency. The work is more evenly distributed among ambulance drivers, and drivers are less likely to get overwhelmed. Health care providers’ acceptability of the ECDC was further facilitated by health workers’ realization that the burden of mobilizing patients for transport, arranging fuel, and accompanying the patient shifted from them to ECDC agents. To our knowledge, there are no previous studies on the role of call and dispatch centers in improving equity in duty allocation.

One barrier to the success of the ECDC and the application was the number of available ambulances. These findings showed that there were too few public ambulances to cover the demand. As demand is increasing, there is a need to advocate for more ambulances for the city to reduce delays in accessing transport and decrease waiting time. In addition, the ambulances lacked basic first aid kits, resuscitation amenities, essential drugs, and oxygen. Reflection by the implementation research team and leadership of the capital city highlighted the need to have EMTs stabilize emergency cases during transfer. The EMTs may improve client satisfaction with the referral process, improve provision of dignified and respectful care, decrease abuse during transfer, and improve patient outcomes.

Another barrier to application use was the Internet connectivity and data availability to the health care providers and drivers. Poor Internet connectivity delayed the input of data into the application, and, as a result, in emergency situations, providers often resorted to calling the ECDC to request transport. For the future, KCCA was negotiating with partner projects within the facilities that have reliable Internet connectivity to support the referral system.

The low level of technological skills of some providers was a barrier to uptake of the application in the initial stages of implementation. For the scale-up of the application, data and technical support should be provided to increase the adoption of the innovation.

### Strengths

The involvement of the city or district leadership from cocreation to implementation and in the dissemination of the findings promoted the acceptability and sustainability of the intervention. The MaNe project enabled sustainability of the interventions after the project ended. For example, district leadership was paying the salaries of the call center staff. However, there is a need to assess the extent to which different participants in the referral/ambulance system have ongoing access to technical assistance, resources, motivation, and linkages. In addition, the MaNe project and KCCA partnership with EMT training institutions enabled the deployment of trainees, and this relieved the human resource workload at the health facilities as nurses and midwives no longer had to accompany the referred patient to the referral facilities.

### Limitations

This study had some limitations. First, this article did not present pre-referral stabilization of the patient, care during the referral, and feedback to the referring facility, which are also important aspects of a well-coordinated referral system. Second, it did not evaluate the effectiveness of the innovations on MNH outcomes. Establishing a relationship between the interventions and MNH outcomes is pertinent to show the continued need for these interventions. Third, implementation during the COVID-19 pandemic overstretched the call center operations beyond MNH. In addition, the pandemic affected observation and face-to-face interactions for data collection. Fourth, the classification of disease conditions by the ECDC team was based on what callers reported, which may not have been accurate. An Internet shutdown during the presidential elections may have affected uptake of the application. Lastly, one of the planned activities of the project in strengthening the referral system was harnessing many private ambulances available in the city. The MaNe project was not able to report the effect of this specific activity because the private ambulances were not yet on board by the time the data were collected. There were delays in the contractual process for their inclusion, and it was finalized at the end of the project.

## CONCLUSIONS

Establishment of the call center and development and deployment of the mobile application for coordinating referrals is feasible. The mobile application improved the coordination of drivers and ambulances and allowed facilities to prepare for and treat cases more efficiently. We recommend it be scaled up to other regions of the country; however, having strong and visionary leadership is key to ensuring ownership, cost-sharing, and sustainability. Finally, there is a need to conduct a thorough study using established sustainability frameworks to understand the gaps in the institutionalization of interventions after the closure of the project.

## Supplementary Material

22-00332-Ononge-Supplement.pdf

## Notes

**Peer Reviewed**

**First published online:** May 18, 2023.
