# Supplementary material for: Strengthening Kampala’s Urban Referral System for Maternal and Newborn Care Through Establishment of an Emergency Call and Dispatch Center
Source: Glob Health Sci Pract. 2023 Jun 21;11(3):e2200332. doi: 10.9745/GHSP-D-22-00332 (PMC10285736; doi:10.9745/GHSP-D-22-00332)
Supplement: 22-00332-Ononge-Supplement.pdf [file 22-00332-Ononge-Supplement.pdf]

THE PROCESS OF CO-DESIGNING A PROJECT WITH EMPATHY, INSIGHTS, AND PROTOTYPING (EIP): THE EXPERIENCE OF THE KAMPALA SLUM MATERNAL NEWBORN HEALTH (MaNe) PROJECT

Authors:
Andrew Magunda[1], Henry Kaula[1], Peter Buyungo[1], Dorothy Balaba[1], Yvonne Mugerwa[1], Okello Daniel Ayen[2], Brett Keller[1]
[1]Population Services International
[2]Kampala Capital City Authority

Introduction and Background:

Population Services International (PSI) and its partner, Kampala Capital City Authority (KCCA), are using an implementation science approach to test innovative interventions addressing demand and supply barriers which impede provision of quality maternal and newborn health (MNH) care in urban slum settings of Kampala City, Uganda. This is being done through the Kampala Slum Maternal Newborn Health (MaNe) project, funded by the United States Agency for International Development (USAID)'s Health Research Program.

The MaNe project used PSI's "Empathy, Insights and Prototypes" (EIP) model, an approach to integrating human centered design (HCD) methods into program design, to structure its interventions. HCD is an approach to interactive systems development that aims to make systems functional by focusing on the users, their needs, and requirements, and by applying human factors, and usability knowledge and techniques11.

PSI's EIP model is an approach to program development that draws on aspects of HCD and other programmatic inputs to design a program. MaNe used this EIP approach to understand the supply and demand side barriers in the MNH market, which currently is failing the urban poor and contributing to poor maternal and newborn health outcomes. The EIP approach MaNe took started with conducting primary and secondary formative research work to understand these MNH market failures.

The project later convened two co-design workshops with key MNH stakeholders which provided an opportunity for a deep dive into the data from the formative research, for stakeholder ideation and development of interventions to address the barriers to MNH.

The project objectives and Theory of Change (TOC) were modified based on outputs from these workshops. The proposed solutions generated through this model were rapidly tested for initial feedback before piloting at a larger scale. In this brief, we describe the process of co-designing MaNe project interventions using the EIP model.

Definition and purpose of each phase of EIP

The EIP model is built on three phases: Empathy, Insights and Prototyping[2].

The Empathy phase:

Empathy is the ability to be aware of, understanding of, and sensitive to another person's feelings and thoughts without having had the same experience. It is also a fundamental cultural value that allows for the development of solutions (concepts, products, services, strategies, interventions) that are both innovative and responsive to actual user needs and desires. The Empathy phase draws on HCD approaches to "immersion". This phase is focused on observing people's behavior.

The Insights phase:

Insights are ideas that help structure our interpretation of patterns in our research findings. In this phase of EIP, the program team uses the experience of the Empathy phase to arrive at insights. These insights ideally offer a new perspective, even if they aren't always new discoveries. They are inspiring and relevant to the challenge we are addressing and often help us explain the 'why' behind a behavior.

PHASE 1

Empathy

Ability to be aware of, and sensitive to others' feelings and thoughts without having had the same experience.

Power of recommendation or experience

Women will use facilities based on experience with them, word of mouth and reccmmendation by friends, family and spouses.

PHASE 2

Insights

Ideas that interpret patterns in our research findings, they offer a new perspective, not necessarily new discoveries.

Trust with the source of information is the strongest basis for driving ANC and newborn care behavior. Women would accept information when passed to them by people they consider to be taking care of their interests.

PHASE 3

Design Challenge

Events, processes and actions that help create better health solutions to identified gaps during the formative phase

How we might leverage experienced mothers & other trusted people like health workers as sources of (correct) information to influence healthy beahviour of peers & especially first time mothers ? How might we use culture as a steppingstone to influence positive health behaviours .

PHASE 4

Ideation

Brainstorm and generate ideas for design of interventions & deciding which ones are worth further development

Women would use health services more if VHTs were equipped with skills and dedicated more time and commitment to the work.

PHASE 5

Prototype

Develop interventions, rapidly test them with audience to get feedback & iterate until a best fit is derived.

Prototype 1: Voluntary Conmmunity Health Worker - CHWs

Voluntary CHWs & peer educators per zone providing MNH messages & birth preparedness to the public

The Prototyping Phase:

Prototyping is the development of interventions, rapidly testing them with the target audience to get feedback, and continuously iterating them until a best fit is derived

The EIP model as applied to the MaNe project:

Above is a pictorial illustration of the five steps of the EIP process applied by the MaNe project team. This illustration uses an example of how we arrived at using voluntary community health workers as a prototype/ intervention to be tested. In the next steps, this brief describes in more detail the activities/ actions undertaken in each EIP step.

Empathy:

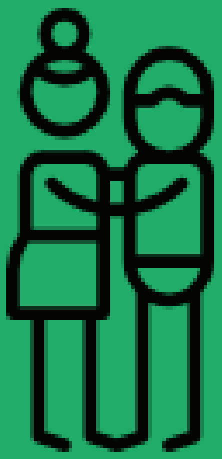

Ability to be aware of, and sensitive to others' feelings and thoughts without having had the same experience.

Empathy refers to the process of connecting with intended users of the intervention; with the aim of comprehending their needs, thoughts, hopes, desires, emotions or motivations.

In its initial year, the MaNe project set out to understand the gaps in the MNH market for Kampala. This was done through four key studies.

These included; 1) a qualitative primary formative study focused on understanding the stakeholder perspectives and experiences on MNH services in Kampala's urban slum communities; 2) a secondary analysis of key data sets (for example DHIS2 data and the Uganda Demographic Health Survey 2016) to understand the state of MNH in Kampala; 3) a scoping review study focused on literature review on implementation of MNH interventions for the urban poor in Sub-Saharan Africa; 4) a health facility assessment on MNH service provision.
Using the evidence from the four studies above about the MNH market failures, MaNe was able to gain a deeper understanding and identify the root causes, priority constraints, supply-side failures, and the socio-ecological failures in the MNH market in Kampala.

Example of a research finding on community health workers during the empathy:
"Power of recommendation/experience – Women will use facilities based on past experience with them, word of mouth and recommendation by friends, family and spouses".

Output:
The results from the four (4) formative research studies formed the basis for deriving/generating key insights at the data interpretation workshop and co-design workshop.

Insights

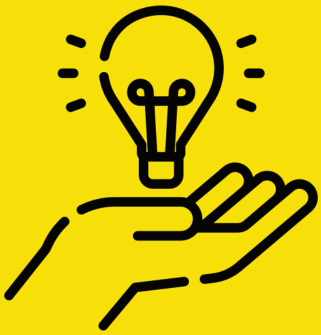

Ideas that interpret patterns in our research findings, they offer a new perspective, not necessarily new discoveries.

Insight development: Insight development involved generating an accurate and intuitive understanding of different issues arising out of the research findings.

It goes beyond a restatement of facts uncovered in the research to understand drivers of health behavior of the urban slum population in Kampala. Our insights, therefore, were a transformation of research findings into a generalized understanding of the situation and how these insights can be used to design solutions for the MNH gaps identified through the research. Data interpretation workshop as part of insight development: The MaNe team used a data interpretation workshop and follow-up internal technical debrief sessions to generate insights from the formative research findings. At the workshop, health experts from Population Services International (PSI), Kampala Capital City Authority (KCCA), KCCA healthcare providers, community representatives including political leaders, as well as researchers from Makerere University School of Public Health (MaKSPH), were brought together to internalize and interpret the findings. They spent a day summarizing the findings from the four major project formative studies.

As a result of this exercise, the project team was able to define, agree on and extract the relevant formative research data to be presented and used in the co-design meeting. They were also able to interpret this data and to glean key insights that would form the basis for generating solutions to health system challenges in Kampala.

Example of insight developed on community health workers:

"Trust with the source of information is the strongest basis for driving ANC and new-born care behavior. Women would accept information when passed to them by people they consider to be taking care of their interest

Output:

A total of twenty (20) key insights were generated from the formative research findings to guide the formulation of design challenges, and ideation at the co-design workshops.

Design Challenge

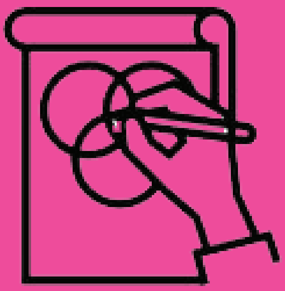

Design challenges are descriptions of events, processes, and actions, delineating or outlining the gap to be addressed

Events, processes and actions that help create better health solutions to identified gaps during the formative phase

The identification and articulation of "design challenges" is the crucial intersection between the insights phase and the start of the ideation phase. Following the data interpretation workshop in which participants identified key insights from the formative research, the MaNe project team spent time articulating the underlying problems/gaps from which the generated insights are derived, thus defining the scope of the desired solutions (prototype/innovations) more clearly.

To create discrete needs that together constitute the overall programmatic challenges, the design challenges were structured in alignment with the key insights. They were generated using *"How might we"* questions to allow flow of ideas for the solutions to the challenges. Different participants at the co-design workshop were put into different groups and assigned different design challenges to ideate.

Example of design challenge on community health workers:

"How might we leverage experienced mothers and other trusted people like health workers as sources of (correct) information to influence healthy behavior of peers and especially first-time mothers? How might we use culture as a stepping-stone to influence positive health behaviors (and correct false beliefs)?"

Output:

The eighteen (18) generated design challenges were used in the next phase of co-design – ideation to articulate actionable solutions that would serve as key MNH interventions.

Ideation

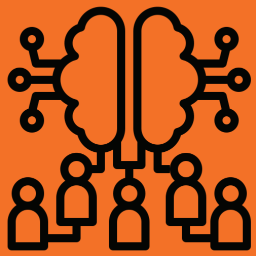

Brainstorm and generate ideas for design of interventions & deciding which ones are worth further development

MaNe initiated ideation to develop ideas for addressing the design challenges arising from the previous phase.

Following the data interpretation workshop in which insights were generated, MaNe convened two co-design workshops with over 180 participants including health sector development partners, community members, and international stakeholders.

The purpose was to engage them in using these insights to propose innovative interventions (generate prototypes) necessary for improving MNH for the urban poor.

The co-design meetings also provided an opportunity for a deeper dive into the data from the empathy phase. The project objectives and Theory of Change were modified based on outputs from the ideation.

In the first phase of the co-design workshop, the facilitators organized participants into diverse groups of stakeholders from different backgrounds, and organizations, and were tasked to come up with solutions to each of the design challenges. Led by a group leader in the brainstorming exercise, the groups addressed one design challenge at a time. They had the liberty to generate as many ideas as possible for each of the design challenges. By tracking each of the ideas using post-it notes, the group members actively listened to what each member had to say so as to build on each other's ideas.

The ideas that were selected for consideration had to generally fit the following criteria: 1) they had to be able to demonstrate health impact by addressing the generated insights and MaNe objectives; 2) they had to be deemed desirable to the target audience; 3) they had to be innovative, feasible, scalable, and sustainable, and able to continue impacting lives beyond the life of the MaNe project.

Example of ideation designed on community health workers:

"Women would use health services more if village health workers were equipped with skills and dedicated more time and commitment to the work"

Output:

A total of 102 ideas were generated from the ideation sessions, which were further refined to formulate prototypes of interventions to be tested.

Prototype

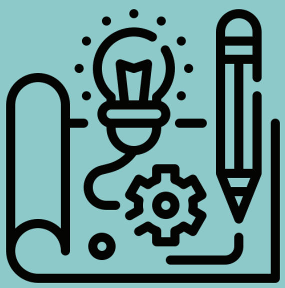

Develop interventions, rapidly test them with audience to get feedback & iterate until a best fit is derived.

Prototyping involves development of low-cost models that can be rapidly tested with the target audience to get feedback, and then iterated until a best fit is derived4

Within the same co-design workshops, the focus then shifted from just generating ideas, to collating initial plans for how to test out those ideas. At the co-design workshops, prototypes were developed for each potential solution generated during the ideation steps, guided by key questions:

1) What would be done, how and when?
2) Who would implement the idea?
3) Which target group?
4) Anticipated challenges and how to overcome them.

Example of a prototype on community health workers:

"Identify & train CHWs & equip them with basic knowledge about MNH & the necessary tools to use to reach mothers"

The second round of testing of prototypes whose features had been found to be acceptable and effective but requiring minor modifications to further increase their desirability and likelihood of effectiveness. It recommended following the evaluation of feedback from the low fidelity testing and making of revisions based on this feedback. Of the 17 prototypes that went through low fidelity testing, 11 did not require high fidelity testing while the other six did. These were re-tested with both providers and community representatives, using similar assessment questions and methods used in the low-fidelity testing.

Prioritization of prototypes was based on feedback and ranking criteria that included; acceptability and desirability, feasibility, cost-effectiveness, scalability, effectiveness, and likelihood/possibility of impact.

Output: A total of 17 prototypes were tested and feedback generated was used to refine and agree on the final prototypes to be piloted and later scaled up as interventions once proved effective.

BELOW IS AN EXAMPLE OF FEEDBACK FROM A DESIGN SPRINT ON THE PROTOTYPE - VOLUNTARY COMMUNITY HEALTH WORKERS.

PROTOTYPE NAME 1: VOLUNTARY COMMUNITY HEALTH WORKER-CHWS

FEATURE 1: Voluntary CHWs per zone/village providing Maternal and New-born messages & birth preparedness to the public

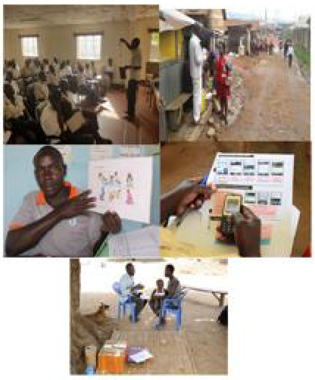

Key field observations/feedback-1st round of testing

Idea is very good;
- CHWs must have lived in the community for at least 5 years.
- Should have extensive knowledge about the residents in the community

Key changes/adjustments/additions for 2nd round of testing

- CHW shall be equipped with information on MNH
- Will be born or resident in the community for at least 5 years
- Good track record in the community e.g not drunkards
- Prioritise those with some position of leadership in the community like LCs, women representatives on village local councils
- These will provide different messaging per category of mothers e.g messages for 1st time mothers shall be different

Piloting Interventions

The MaNe project is currently piloting the prioritized interventions. In preparation for piloting, it is helpful to have a written description of each prototype based on the feedback and refining process of the prototypes, into an intervention concept document. The document should include the name and description of the intervention, names of different features and elements of the intervention, the rationale of the elements (why), the physical and information materials to be used in the feature, source of the material. It should also describe the process activities to be used in the intervention and any enabling activities, expertise of who will do the execution, modes of delivery, location, number of times the intervention will be delivered, as well as the session schedules, duration, intensity and period.

Lessons Learned from the MaNe EIP process

Utilization of the EIP model ***enabled the MaNe team to generate insights and collaboratively design intervention packages*** or delivery mechanisms based on a better understanding of the urban poor and dynamics of service provision within Kampala's slums.

Having a diversity of co-creators means that varied experiences, ideas, and expertise are tapped into to inform the intervention design and development. This generally included the following: Non-governmental Organizations (NGOs), multilateral organizations, academic institutions, Ministry of Health, Kampala Capital City Authority, public and private health providers, national associations, and community members.

Using participant-based approaches in the process of co-design ensures that participants become agents of the co-creation process and this facilitates ownership of generated ideas. Some of the examples of participant approaches used include gallery walks and break-out sessions. The gallery walks, which had sprint questions and quotations, enabled participants to appreciate the experiences of the urban poor mothers based on the different responses from the interviews. The break-out group sessions helped to improve participation, especially by the members of the community with common views. They also created an opportunity to engage the different stakeholders in targeted ways to maximize productivity.

In a co-design workshop that attracts different stakeholders at different levels of the health care systems, ***many participants did not speak the same language.*** The co-design workshop was conducted in both English and Luganda, which enabled participants, especially representatives from the urban poor community, to follow every session and to comfortably express themselves and their ideas.

In the process of co-creation of interventions, while the great diversity of participants can enrich the scope of proposed solutions to critical health system challenges, ***working with such many participants can affect the quality of the EIP process since managing them turned out to be a complex process.*** Parallel/break-out sessions were used to better handle the large numbers. It should be noted however, that the diversity of the participants resulted in generation of a wider spectrum of solutions.

Using the EIP model in the design of key development interventions requires enough time, robust guidance, and several technical team members for critical processes.

Some phases of the model which are a critical path for the EIP process require more time than others. For example, brainstorming and generating ideas for the design of interventions and determining which ones require further development. It was important not to push ahead to the next phase of co-designing at points when the participants and facilitation team needed more time to deliberate and negotiate. Such critical processes include prioritization of interventions for consideration. This required sufficient time to discuss what should be prototyped or what should be dropped from the prototype list or features.

Additionally, the four-day co- creation workshop in the EIP process could have benefited from more than just the two separate stakeholder engagement workshops. This is because an additional workshop would give designers an opportunity to spend focused time between the process of ideation and prototype development. Having these as separately organized workshops would contribute to better prioritization of solutions and having a better-defined scope of interventions to maximize impact.

It is very critical to provide detailed guidance on some key areas of the EIP process, especially on the prioritization of issues and solutions for complex interventions. Lack of clear, standard criteria for prioritizing problems or solutions can create time, content, scope, and quality constraints in the process.

Prioritization of solutions and prototypes as well as iteration should be done by more than one individual/team to minimize subjectivity and selection bias. Outputs of the different individuals/teams would then be compared for reliability and a final list objectively developed. Additionally, a clear selection criterion should be developed to guide participants during and after the workshop. Strategies and tools to augment the steps between insights development and piloting are critical points.

References

1. https://en.wikipedia.org/wiki/Human-centered_design

2. O'Cathain1 A, Croot L, Sworn K, Duncan E, Rousseau N, Turner K, Yardley L and Hoddinott P. 2019. Taxonomy of approaches to developing interventions to improve health: a systematic methods overview. Pilot and Feasibility, 5:41

3. Norman D. 2013. The design of everyday things: Revised and expanded edition

4. Hawkins J, Madden K, Fletcher A, Midgley L, Grant A, Cox G, Moore L, Campbell R, Murphy S, Bonell C and White J. Development of a framework for the co-production and prototyping of public health interventions. BMC Public Health (2017) 17:689BMC Public Health (2017) 17:689

This learning brief is made possible by the generous support of the American people through the United States Agency for International Development (USAID) under cooperative agreement number 7200AA18CA0005. The contents of this presentation are the responsibility of Population Services International (PSI) and do not necessarily reflect the views of USAID or the United States Government.
